# From conventional probiotics to precision nutrition: host-specific probiotics in canine and feline functional foods

**DOI:** 10.3389/fvets.2026.1881134

**Published:** 2026-07-21

**Authors:** Zhihao Wang, Wenjing Wang, Pei Zhang, Jie Luo, Yi Zhang, Qingwen Yang

**Affiliations:** 1 Laboratory of Veterinary Pharmacology, Department of Animal Science and Technology, Chongqing Three Gorges Vocational College, Chongqing, China; 2 Heilongjiang Center for Animal Disease Prevention and Control, Heilongjiang, China; 3 Department of Agriculture and Animal Husbandry, Ganzi Vocational College, Sichuan, China; 4 Department of Economics and Management, Chongqing Three Gorges Vocational College, Chongqing, China

**Keywords:** functional pet food, host specificity, pets, probiotics, stabilization technologies

## Abstract

Probiotics are widely used in companion animal nutrition due to their roles in maintaining gut health, regulating the microbiota, and supporting metabolic homeostasis. Here, we summarize recent advances in the application of probiotics in functional foods for dogs and cats. We outline the strain resources, functional mechanisms, and stabilization technologies of probiotics, with emphasis on their effects on gut barrier function, immune regulation, and metabolic health. In addition, the current challenges, including insufficient host specificity, a lack of rigorous clinical trials, and poor processing stability are discussed. Future directions are proposed for the development of precision probiotics for companion animals. This review provides a theoretical basis for the rational design and standardized application of probiotics in the pet food industry.

## 1 Introduction

With the rapid development of the global companion animal industry, pets have transitioned from being mere “companions” to becoming integral “family members” in human households (1, 2). The growing emphasis on pet health has driven the pet food industry to evolve from basic nutritional provision to precise health interventions, with functional foods emerging as the key driver of industry growth (3). Probiotics, defined as live microorganisms that confer health benefits to the host when administered in adequate amounts, have become the most widely used functional ingredients in functional pet foods due to their advantages of safety, non-toxicity, and multi-target health regulation (4, 5). Research indicates that probiotics effectively alleviate gastrointestinal disorders in dogs and cats, including diarrhea, constipation, and inflammatory bowel disease, while also alleviating clinical symptoms such as chronic kidney disease, obesity, allergies, and oral malodor (6–10). Furthermore, they help reduce reliance on antibiotics and mitigate associated health risks (11).

Currently, the forms of pet probiotic products have expanded from single powder supplements to a diverse functional food system integrated into main diets, snacks, wet foods, chewable tablets, with the market scale continuing to grow (12). Nevertheless, probiotics incorporated into functional pet foods still face critical limitations, including insufficient host adaptation, ambiguous functional mechanisms, and low viable cell survival rates during industrial processing (13, 14). Here, we review the research progress in the application of probiotics in functional pet foods, summarize the technological bottlenecks and development trends in the industry, and provide theoretical support for the research and development of probiotics in functional pet foods as well as the industrial development.

## 2 Host specificity and probiotic strain types in functional pet foods

The biological functions of probiotics exhibit significant strain-specificity and host-specificity, with their interventional effects influenced by both the genetic background of the strain and the physiological environment of the host. Consequently, the selection of probiotics specifically for companion animals must adhere to rigorous criteria (15). Currently, the probiotics approved for use in pet foods globally primarily include the genera *Lactobacillus*, *Bifidobacterium*, *Bacillus*, *Enterococcus*, and *Saccharomyces* (16, 17). Strains from these different genera exhibit distinct functional differences in metabolic characteristics, mechanisms of action, and clinical applications (18).

### 2.1 Host specificity

#### 2.1.1 Canine and feline gut microbiota

As carnivores, dogs and cats have a shorter intestinal tract and thicker intestinal wall compared with humans and other livestock (19). Their energy acquisition primarily depends on the direct digestion and absorption of dietary protein and fat rather than microbial fermentation, a physiological characteristic that fundamentally shapes their unique gut microecological structure (20). The gut microbiota of dogs and cats consists of aerobic, facultative anaerobic, and obligate anaerobic bacteria, with abundance and diversity exhibiting a significant gradient increase along the gastrointestinal tract where microbial load is lowest in the stomach and gradually increases from the small intestine to the colon (21). In healthy dogs and cats, the gastric microbiota is dominated by the genera *Helicobacter* and *Lactobacillus*, while the dominant phyla in the intestine are *Firmicutes*, *Proteobacteria*, *Bacteroidetes*, *Fusobacteria*, and *Actinobacteria*. Distinct variations exist between the intestinal microbiota of dogs and cats. Specifically, the dominant flora in the duodenum and jejunum of dogs primarily comprises *fungi*, *Bacteroides*, *Clostridium*, *Bifidobacterium*, and *Lactobacillus*, whereas the feline duodenum is characterized by *Pasteurella*, *Bacteroides*, *fungi*, and *Clostridium*, with the jejunum dominated by *Enterococcus*, *Streptococcus*, and *Lactobacillus* (22, 23). Regarding fecal microbiota composition, dogs are characterized by *Clostridium*, *Enterococcus*, and *fungi*, while feline feces exhibit higher abundances of *Enterococcus*, *Streptococcus*, *Lactobacillus*, and *Clostridium* (24). Domestic cats are obligate carnivores that require high-protein diets, whereas dogs possess the metabolic capacity to digest, absorb, and metabolize a broader range of carbohydrates (25). Consequently, differences in dietary composition are considered a significant factor contributing to the structural divergence of the gut microbiota between these two species.

#### 2.1.2 Host specificity of probiotics

The biological functions of probiotics are tightly associated with their host specificity. Accumulating evidence indicates that, compared with strains isolated from other species, host-derived probiotics exert more pronounced beneficial effects in their native hosts. For instance, compared with chicken-derived strains of the same genus, *E. faecalis* and *E. faecium* isolated from canine sources demonstrated higher adhesion to canine duodenal mucosa. Furthermore, bacterial surface hydrophobicity assays confirmed that the canine *E. faecalis* isolate possessed the highest level of hydrophobicity (26). In another study, *L. acidophilus LAB20,* isolated from canine jejunum, demonstrated markedly greater adhesion to canine colonic mucus than to porcine and human colonic mucus; this strain also displayed anti-inflammatory activity, as evidenced by its capacity to attenuate lipopolysaccharide (LPS)-induced interleukin-8 (IL-8) production in intestinal epithelial cells. In contrast, the human-derived *L. rhamnosus GG* strain showed superior adhesion to human colonic mucus compared with porcine and canine colonic mucus (27). A characterization study of the gut microbiome in 238 pet dogs and cats revealed significant inter-species microbial differences between dogs and cats. Moreover, substantial inter-individual variations in bacterial composition were observed within canine breeds, a divergence that was particularly pronounced at finer taxonomic levels. The study also suggested that geographic location and body weight may serve as critical covariates influencing the community structure of the canine gut microbiome (28). Collectively, these findings suggest that probiotic efficacy may vary not only across different host species but also among different breeds or geographic populations within the same species, potentially driven by variations in their microecological backgrounds. This underscores the necessity of constructing dedicated biobanks for dog and cat microbiomes, thereby enabling the targeted isolation of probiotic strains adapted to specific demographic and geographic profiles. Beyond isolating candidate probiotic strains from the intestinal tract and feces of dogs and cats, the screening of strains with robust probiotic activity is equally indispensable for practical application. This screening typically encompasses *in vitro* assessments of acid tolerance, bile salt tolerance, surface hydrophobicity, antioxidant capacity, and antimicrobial activity, along with *in vivo* evaluations of their protective effects on the host intestinal barrier, anti-inflammatory activity, and immunomodulatory functions (5, 13).

### 2.2 Probiotic strain types

#### 2.2.1 Lactobacillus

The genus *Lactobacillus* belongs to the Gram-positive, facultatively anaerobic bacteria (29). Common strains incorporated into functional pet foods include *L. plantarum*, *L. paracasei*, *L. acidophilus*, *L. reuteri*, and *L. rhamnosus*. Among these, *L. plantarum* and *L. paracasei*, mostly isolated from fermented foods, exhibit lower intestinal tolerance compared to *L. acidophilus*, which may explain their less frequent application in pet foods (16). Through carbohydrate fermentation, lactobacilli produce lactic acid to lower intestinal pH and restrict pathogen growth, while also exerting distinct immunomodulatory, anti-inflammatory, and metabolic-regulatory functions (30–32).

#### 2.2.2 Bifidobacterium

*Bifidobacterium* is a genus of Gram-positive, strictly anaerobic bacteria, with commonly utilized strains including *B. animalis*, *B. longum*, and *B. breve* (33). *Bifidobacterium* can ferment dietary fiber to produce short-chain fatty acids, which provide energy to intestinal epithelial cells, while also improving fecal quality, enhancing immune function, regulating nutrient metabolism, and promoting intestinal barrier repair (34, 35). However, *Bifidobacterium* exhibits high sensitivity to oxygen and temperature; exposure to high heat and oxygen during processing often leads to cell death and poor viability (36). Consequently, their application is primarily restricted to low-temperature treats, wet foods, and powdered supplements, frequently necessitating the use of stabilization technologies. Notably, Wang (37) demonstrated that a synbiotic formulation comprising heat-inactivated *B. longum CECT-7347* and prebiotics significantly alleviated gut dysbiosis induced by dietary transitions in cats, achieving efficacy comparable to that of viable probiotics. These findings offer a novel avenue for the broader application of *Bifidobacterium*.

#### 2.2.3 Bacillus

*Bacillus* is a genus of Gram-positive, aerobic or facultatively anaerobic bacteria, with commonly utilized strains including *B. subtilis*, *B. coagulans*, and *B. amyloliquefaciens* (38). A distinguishing feature of this genus is the ability to form endospores, which confer exceptional resistance to high temperatures, gastric acidity, and prolonged storage (39). These attributes render them uniquely compatible with the high-temperature extrusion processes employed in pet food manufacturing, as they maintain high viability even after short-term exposure to 120 °C. Consequently, *Bacillus* species represent the most widely utilized group of probiotics in commercial pet staple foods. Upon germination within the canine and feline gastrointestinal tract, these bacteria rapidly deplete intraluminal oxygen, thereby establishing an anaerobic environment that facilitates the proliferation of commensal anaerobes, such as *Lactobacillus* and *Bifidobacterium* (40). Furthermore, they secrete exogenous enzymes, including proteases and amylases, which enhance the digestibility and absorption of nutrients, significantly improve fecal morphology, and reduce the concentration of putrefactive toxins within the intestine (41).

#### 2.2.4 Enterococcus

*Enterococcus*, a Gram-positive coccus generally regarded as safe when properly screened, exhibits robust tolerance to acidic conditions and bile salts. It is frequently utilized as an adjunctive intervention for diarrhea in pets due to its capacity to rapidly alleviate symptoms and enhance intestinal mucosal immunity (42). However, *Enterococci* are also known to harbor clinically significant antimicrobial resistance genes, such as vanA and vanB (vancomycin resistance) and optrA and cfr (linezolid resistance), which can be horizontally transferred to other pathogens via plasmids (43). Consequently, rigorous screening for antibiotic resistance is imperative for *Enterococcus* strains intended for probiotic applications.

#### 2.2.5 Saccharomyces

*Saccharomyces* is a class of fungal probiotics, with *Saccharomyces cerevisiae* and *Saccharomyces boulardii* being the most widely utilized species (44, 45). Characterized by their intrinsic resistance to antibiotics and gastric acidity, these yeasts are effective in ameliorating antibiotic-associated diarrhea in dogs and cats, modulating gut microbiota composition, and reducing the duration of diarrheal episodes (46, 47).

## 3 Mechanisms of probiotic action

### 3.1 Modulation of gut health

Regulation of intestinal health is the core and most fundamental biological function of probiotics, as well as their primary application scenario in functional pet foods. Traditionally, antibiotics have been commonly employed for the treatment of acute and chronic gastrointestinal diseases in dogs and cats. However, as modern genomic technologies have revealed the profoundly negative impacts of antibiotics on the gastrointestinal microbiota, coupled with the rapid global spread of antimicrobial-resistant bacteria, there is an increasingly urgent need for safe and effective alternatives to antibiotics in small animal clinical practice. Existing studies have confirmed that certain probiotics can adhere to intestinal epithelial cells through their own structural components (such as surface proteins) and metabolites, thereby competitively blocking the adhesion sites of pathogenic bacteria, sequestering nutrient sources, and inhibiting their growth and proliferation (48). Probiotics protect intestinal health by producing bacteriostatic and bactericidal active substances including organic acids and bacteriocins via metabolic pathways (49). For example, *Ligilactobacillus murinus ZQL11* isolated from canine feces exhibits remarkable *in vitro* antibacterial activity against *Escherichia coli*, *Salmonella* spp. and *Staphylococcus aureus* (50). In addition, some probiotics can promote the production of short-chain fatty acids (SCFAs) including acetic acid, propionic acid and butyric acid. As the primary energy substrate for intestinal epithelial cells, butyric acid can repair the intestinal mechanical barrier and immune barrier, and attenuate intestinal inflammatory responses (51). For instance, a study supplementing feline diets with *Saccharomyces boulardii* and *Pediococcus acidilactici* demonstrated that, compared with the control group, probiotic supplementation significantly increased the concentrations of butyrate and total SCFAs in feces, while concurrently reducing inflammatory levels (52). Collectively, probiotics have demonstrated the capacity to effectively improve gastrointestinal function, modulate the gut microbiome, and alleviate common clinical manifestations such as diarrhea and constipation in canines and felines, thereby exhibiting promising prospects as alternative therapies to antibiotics.

### 3.2 Regulation of nutrient metabolism

The global prevalence of obesity in dogs and cats has been increasing annually (53). Obesity can induce a series of complications including diabetes mellitus and hyperlipidemia, which significantly shorten the lifespan of companion animals (54). Probiotics can produce bile salt hydrolase (BSH) to hydrolyze conjugated bile acids, thereby promoting the hepatic synthesis of bile acids from cholesterol and reducing serum cholesterol levels (55). In addition, probiotics can delay the degradation and absorption of carbohydrates in the intestinal tract and reduce the postprandial blood glucose peak by inhibiting the activities of *α*-amylase and α-glucosidase (56). A study in which *L. plantarum L-27-2* and *Pediococcus lactis L-14-1*, isolated from feline feces, were separately administered to cats demonstrated that both strains significantly reduced serum levels of triglyceride and low-density lipoprotein cholesterol, while significantly increasing high-density lipoprotein cholesterol levels (57). Although probiotic strains isolated from dogs and cats have been shown to possess hypoglycemic and hypolipidemic properties, the specific mechanisms underlying these effects within the canine and feline hosts remain to be fully elucidated (58).

### 3.3 Adjuvant therapy for chronic kidney disease

Chronic kidney disease (CKD) is a highly prevalent progressive disease in elderly cats, for which no curative clinical treatment is currently available, with the core therapeutic principle focusing on controlling clinical symptoms and slowing disease progression (59). The proposal of the “gut-renal axis” theory has provided a novel research direction and therapeutic target for the nutritional intervention of CKD (60). Impaired renal filtration and excretion function leads to the accumulation of uremic toxins in the body, while intestinal dysbiosis is the core driver of the production and accumulation of gut-derived uremic toxins (GDUTs). Probiotics can reduce the production and intestinal absorption of GDUTs via targeted regulation of intestinal microbiota structure, thereby delaying the progression of CKD (61). In a pioneering multi-omics study on probiotic intervention in feline CKD, Huang (62) confirmed that supplementation with *Lactobacillu*s *mix* (Lm) ameliorated host intestinal dysbiosis and modulated microbial functions related to the production of uremic toxins and SCFAs. This intervention effect was closely correlated with favorable shifts in the host systemic metabolic profile, as evidenced by significantly reduced levels of indoxyl sulfate (IS), p-cresyl sulfate (PCS) and phenyl sulfate (PS), along with elevated SCFAs concentrations.

### 3.4 Immunomodulation

The intestinal tract is the largest immune organ in the body, with over 70% of immune cells residing in the intestinal mucosal tissue (63). Probiotics can modulate systemic immune function by regulating gut mucosal immunity, thereby enhancing the host’s defense against pathogenic microorganisms. For instance, in murine models, *B. subtilis* intervention can significantly enhance the phagocytic function of the mononuclear phagocyte system (MPS) and the cytotoxicity of natural killer (NK) cells, while increasing the proportion of T cells secreting interferon-*γ* (IFN-γ) (64). Similarly, studies in companion animals have demonstrated that *Bifidobacterium* and *L. plantarum* can repair the intestinal mechanical barrier by reducing plasma D-lactic acid levels and diamine oxidase activity, as well as increasing the abundance of beneficial bacteria in the gastrointestinal tract, thereby significantly upregulating serum immunoglobulin A (IgA) levels (34). Furthermore, *L. rhamnosus* not only increases the number of secretory immunoglobulin A (sIgA)-positive cells in the intestinal mucosa and stimulates the secretion of local intestinal interferons, but also promotes the transport of antigens to lymphocytes in the intestinal lamina propria, thereby enhancing the antigen uptake and presentation capacity of Peyer’s patches (65). These findings indicate that distinct probiotic strains modulate host immunity through diverse and often complementary mechanisms, suggesting that their combined application may exert synergistic effects, thereby more effectively enhancing their anti-inflammatory and immunomodulatory functions (66). A study evaluating the therapeutic efficacy of *Enterococcus faecium Kimate-X* and *L. plantarum Kimate-F* in murine and canine colitis models demonstrated that compared with the single-strain groups, the combination group exhibited greater reductions in serum lipopolysaccharide (LPS) and tumor necrosis factor-*α* (TNF-α) levels, a more pronounced increase in interleukin-10 (IL-10) levels, and markedly attenuated intestinal mucosal injury. Metagenomic analysis of canine fecal samples further revealed that the combination group significantly improved the composition and structure of the gut microbiota, with notable enrichment in key metabolic pathways such as amino acid metabolism, carbohydrate metabolism, and lipid metabolism, indicating a synergistic effect between the two probiotic strains (67). Although the aforementioned findings suggest that multi-strain probiotics may be superior to single strains in enhancing host immunity and alleviating inflammation, the precise mechanisms mediating this synergistic effect remain unclear. Currently, most multi-strain probiotic preparations are formulated by simply combining strains with demonstrated probiotic potential from different genera, without a rational design that accounts for inter-strain interactions. Consequently, which specific combinations yield synergistic benefits and which result in antagonistic or inhibitory effects warrants further systematic investigation.

### 3.5 Improvement of oral and skin health

Oral diseases and dermatitis are highly prevalent among companion animals under domestic feeding conditions (68, 69). Consequently, the application of probiotics in the management of pet oral health and the treatment of dermatitis has garnered increasing attention. *L. acidophilus MJCD175*, isolated from canine feces, exhibits significant inhibitory activity against oral pathogens, including *Porphyromonas gingivalis* (70). Furthermore, a complex probiotic formulation consisting of *B. animalis HN019*, *L. acidophilus NCFM*, and *L. casei LC-11* can regulate the oral microbiota of cats by supporting beneficial or commensal bacteria and inhibiting oral pathogens, showing potential in improving feline oral health (71). Additionally, *Lactococcus cremoris* and *Lacticaseibacillus paracasei* ameliorate clinical symptoms and alleviate pruritus in dogs with atopic dermatitis by modulating immune parameters, demonstrating their potential therapeutic efficacy (72).

## 4 Stabilization technologies of probiotics in functional pet foods

The production of pet food predominantly relies on extrusion technology, a process characterized by extreme physical conditions including high temperature, high pressure, high shear stress, and rapid moisture evaporation (73). These parameters pose severe challenges to the viability of non-spore-forming probiotics, such as *Lactobacillus* and *Bifidobacterium* species, acting as primary stressors that lead to cellular inactivation. Furthermore, the harsh gastrointestinal environment, characterized by low pH and the presence of digestive enzymes, further reduces the survival of lactic acid bacteria. Notably, the viable cell count directly determines the functional efficacy of the final product (74). Guidelines established by the Food and Agriculture Organization (FAO) and the World Health Organization (WHO) stipulate that probiotic strains must survive transit through the upper gastrointestinal tract to exert health-promoting effects at their target sites, regardless of the delivery vehicle (75). Consequently, functional foods containing lactic acid bacteria are generally required to maintain a minimum effective dose of ≥10^6^ CFU/g throughout their shelf life (76). To address these challenges, the industry has developed various stabilization technologies designed to significantly enhance probiotic survival and intestinal delivery efficiency, thereby ensuring compatibility with the processing requirements of diverse functional pet foods (73, 77).

### 4.1 Microencapsulation technology

Microencapsulation technology establishes a physical barrier around probiotic cells, effectively shielding them from environmental stressors such as high temperatures, oxygen, gastric acid, and bile salts (73). This protection significantly enhances strain stability during processing and storage while improving colonization efficiency in the intestinal tract. Commonly used wall materials in this field include biopolymers such as sodium alginate, chitosan, whey protein, and pectin. Choudhary (78) employed sodium alginate and chitosan to create a double-layered encapsulation for *Streptococcus salivarius LAB813*, constructing a reinforced protective shell. Compared to unencapsulated and single-layer controls, the double-layered strain exhibited superior antimicrobial activity across a broad temperature range of 4 °C to 68 °C. Furthermore, the microcapsule formulation maintained its antimicrobial efficacy after 28 days of storage at 4 °C. Guo (79) developed a microcapsule carrier featuring a solid-oil–water emulsion structure, which effectively mitigated the cytotoxicity of antioxidants toward the probiotics. This structure allowed the strain to achieve nearly 100% survival following pasteurization. Additionally, this microencapsulation strategy significantly enhanced the tolerance of probiotics to simulated gastric and intestinal fluids, demonstrating marked advantages in improving probiotic stability under harsh environmental conditions.

### 4.2 Spore formulation and lyophilization protection

Species within the genera *Bacillus* (e.g., *Bacillus subtilis*, *Bacillus coagulans*) and *Clostridium* (e.g., *Clostridium butyricum*) possess the capability to form endospores (80). This biological characteristic confers robust tolerance against thermal stress, desiccation, and acidic environments, rendering them ideal candidates for food additive applications (81). However, the precise regulation of spore germination efficiency *in vivo* remains a significant challenge. Recent studies have established a “spore-xylose” conjugate system by combining *C. butyricum* with chemically modified prebiotic xylose to achieve functional synergy. Upon entering the intestinal tract, the xylose component is fermented by *C. butyricum* under anaerobic conditions, leading to the production of SCFAs that exert beneficial effects on intestinal epithelial cells. Furthermore, this system effectively modulates the gut microbiota composition, significantly increasing the relative abundance of SCFA-producing bacteria and enhancing overall microbial diversity (82).

Lyophilization technology is a critical process for the preparation of solid probiotic formulations. By significantly reducing moisture content, it induces cellular metabolism to enter a dormant state, thereby effectively maintaining the biological activity and long-term storage stability of probiotic strains (83). However, osmotic pressure changes and ice crystal formation during the lyophilization process can induce stress responses, such as cell membrane damage and intracellular water loss. To address such damage, the addition of lyoprotectants, including whey protein, trehalose, sucrose, and ascorbic acid, serves as an effective protective strategy (84). Chen (85) indicated that vacuum freeze-drying significantly reduces the cell adhesion ability and survival rate of *L. plantarum* 67, whereas lyoprotectants can significantly improve the adhesion performance and survival ability of this strain after freeze-drying by regulating the expression of key proteins and metabolic pathways related to adhesion and vitality.

### 4.3 Synbiotics and postbiotics

Synbiotics are defined as composite preparations consisting of probiotics and prebiotics. Prebiotics serve as specific nutritional substrates that promote the colonization and proliferation of probiotics in the gastrointestinal tract of pets, thereby enhancing their efficacy, while also exerting a protective effect on probiotic strains during processing and storage, thus improving their stability (86). Kawano (87) administered a synbiotic formulation comprising *Lactococcus lactis M-1* and the prebiotic trisaccharide kestose to canines with atopic dermatitis. The results demonstrated a significant amelioration of clinical symptoms in the synbiotic-treated group. In addition, Gramenzi (88) demonstrated that the concomitant application of *Limosilactobacillus reuteri* and canine dietary supplements yields significant benefits in promoting intestinal health and enhancing microbial diversity.

The International Scientific Association for Probiotics and Prebiotics (ISAPP) defines postbiotics as preparations of inanimate microorganisms and/or their components that confer health benefits to the host (89). Postbiotics encompass inactivated probiotic cells, cellular components, and metabolites. In contrast to live probiotics, inactivated cell preparations eliminate the risk of infection and horizontal transfer of antibiotic resistance genes, while also exhibiting lower requirements for processing and storage conditions (90). Notably, these non-viable agents can exert probiotic effects independent of viability; however, the extent to which their biological activity is equivalent to that of live microorganisms requires further validation. Sordillo (91) found that a novel postbiotic could significantly reduce the level of volatile sulfur compounds in the oral cavity of dogs, thereby effectively alleviating halitosis symptoms. Furthermore, Wang (37) demonstrated that dietary supplementation with heat-inactivated *Bifidobacterium* in felines contributes to the maintenance of gastrointestinal homeostasis and enhanced immune function.

## 5 Challenges and prospects

The intestinal microecology of dogs and cats exhibits significant species-specific characteristics, and their microbial community structure, metabolic function profile, and antibiotic resistance gene features are significantly different from those of humans and other animals (20). However, strains utilized in currently marketed pet probiotic preparations are mainly isolated from humans, fermented foods, or the intestines of livestock, rather than from the endogenous microbiota of dogs and cats. These allochthonous strains inherently suffer from limitations such as low host adaptability, poor intestinal colonization efficiency, and ill-defined probiotic efficacy (92). Concurrently, the strain combinations in existing products show high uniformity, lacking precision formulations tailored to different age stages and pathological conditions, which fails to meet the clinical demand for refined pet health management. Although current research has focused on isolating host-derived probiotics from the milk and feces of dogs and cats, most existing studies are limited to *in vitro* evaluation of the basic probiotic properties of the strains, such as intestinal barrier regulation and diarrhea control (13, 93, 94). The screening and systematic research of specific functional strains for high-incidence chronic diseases in dogs and cats, such as chronic kidney disease, atopic dermatitis, and obesity-induced glucose and lipid metabolism disorders, are still insufficient. Furthermore, most existing evidence is derived from *in vitro* experiments, mouse animal models, or small-sample preliminary trials, lacking support from large-sample clinical trial data on dogs and cats (93). Although existing theories suggest that probiotics exert modulatory effects through multisystem pathways such as the “gut–kidney axis,” “gut–skin axis,” and “gut–brain axis,” related mechanistic studies are still primarily based on animal models, and research on precise molecular pathways and therapeutic targets in pets remains markedly insufficient (95, 96). Meanwhile, there is a global lack of specific regulations and technical standards for pet probiotics, with no unified guidelines for strain safety evaluation, viable count detection, and functional claim specifications.

Future research should focus on large-scale screening of probiotic strains originating from dogs and cats, integrating multi-omics technologies such as metagenomics and transcriptomics to systematically dissect strain host adaptability and functional characteristics, and developing proprietary probiotic strains specifically designed for pets, thereby fundamentally addressing the issues of low colonization efficiency and unstable efficacy associated with general-purpose strains (92). Meanwhile, targeted strain formulations should be developed according to different age stages and various disease conditions (e.g., chronic kidney disease, obesity, atopic dermatitis) to achieve precise nutritional intervention. Furthermore, there is an urgent need to conduct large-sample, long-term host animal clinical trials to verify the actual effects of probiotics in regulating gut health and preventing chronic diseases, and to establish standardized clinical evaluation systems to ensure the scientific validity and reliability of research outcomes (97). Regarding strain combinations, efforts should be devoted to exploring the synergistic mechanisms among multiple strains, selecting functionally complementary strain combinations to enhance the overall health-regulating effects of probiotic formulations (98). Concurrently, global coordination of regulations and standardization of standards for pet probiotics should be promoted, clarifying core requirements such as strain identification, viable count labeling, storage conditions, and health claims. Drawing on internationally accepted guidelines, such as European Commission Regulation (EC) No 429/2008, Guidance on the assessment of the safety of feed additives for the environment (99), and EFSA statement on the requirements for whole genome sequence analysis of microorganisms intentionally used in the food chain (100), and taking into account the unique physiological characteristics of pets, a specific regulatory framework should be established to regulate the market and raise the overall development level of the industry (15, 76, 99, 100). Finally, it is of practical significance to conduct scientific outreach among pet owners to raise their awareness of the value of species-specific probiotic products. For instance, formulating probiotics into highly palatable delivery forms, such as cat treat sticks and staple diets, can effectively improve owners’ compliance with regular administration.

## 6 Conclusion

The application of probiotics in functional pet foods has emerged as a focal point of research within the global field of animal nutrition. Existing studies have shown that probiotics can significantly improve the intestinal health of dogs and cats, alleviate diarrhea, and play a positive role in the auxiliary management of obesity and certain chronic diseases through mechanisms such as regulating intestinal flora, enhancing intestinal barrier function, producing SCFAs, and regulating immune responses. The application of technologies such as microencapsulation, spore-forming strains, synbiotics, and postbiotics provides effective strategies for ensuring the viability of probiotics during the processing and storage of pet foods. Nevertheless, research on probiotics for dogs and cats is still relatively lagging behind, with the strain-specific mechanisms of action not yet fully elucidated and long-term safety data still insufficient, which restricts the in-depth development of this field. Future efforts should focus on the screening of host-derived strains, the development of functional probiotics, the investigation of probiotic mechanisms via multi-omics approaches, and the execution of long-term clinical trials. Concurrently, there is a need to promote the standardization of relevant global regulations. With the continuous growth in pet owner demand for functional foods and ongoing advancements in biotechnology and food engineering, the application of probiotics in the realm of pet nutrition is poised for a broader horizon and ready to make significant contributions to the health and well-being of dogs and cats worldwide.
